# MicroRNA Promoter Identification in *Arabidopsis* Using Multiple Histone Markers

**DOI:** 10.1155/2015/861402

**Published:** 2015-09-03

**Authors:** Yuming Zhao, Fang Wang, Liran Juan

**Affiliations:** ^1^State Key Laboratory of Tree Genetics and Breeding, Northeast Forestry University, Harbin, Heilongjiang 150001, China; ^2^Information and Computer Engineering College, Northeast Forestry University, Harbin, Heilongjiang 150001, China; ^3^School of Computer Science and Technology, Harbin Institute of Technology, Harbin, Heilongjiang 150001, China

## Abstract

A microRNA is a small noncoding RNA molecule, which functions in RNA silencing and posttranscriptional regulation of gene expression. To understand the mechanism of the activation of microRNA genes, the location of promoter regions driving their expression is required to be annotated precisely. Only a fraction of microRNA genes have confirmed transcription start sites (TSSs), which hinders our understanding of the transcription factor binding events. With the development of the next generation sequencing technology, the chromatin states can be inferred precisely by virtue of a combination of specific histone modifications. Using the genome-wide profiles of nine histone markers including H3K4me2, H3K4me3, H3K9Ac, H3K9me2, H3K18Ac, H3K27me1, H3K27me3, H3K36me2, and H3K36me3, we developed a computational strategy to identify the promoter regions of most microRNA genes in *Arabidopsis*, based upon the assumption that the distribution of histone markers around the TSSs of microRNA genes is similar to the TSSs of protein coding genes. Among 298 miRNA genes, our model identified 42 independent miRNA TSSs and 132 miRNA TSSs, which are located in the promoters of upstream genes. The identification of promoters will provide better understanding of microRNA regulation and can play an important role in the study of diseases at genetic level.

## 1. Introduction

MicroRNAs (miRNAs) are small (~22 nucleotides) noncoding RNAs, which are processed to ~70-nucleotide precursors and subsequently to the mature form by endonucleases [1, 2]. miRNAs are disseminated throughout the genome. They can be found in intergenic regions, intronic regions of protein coding genes, or intronic and exonic regions of noncoding RNAs. They have many regulatory functions in complex organisms. It is known that a single miRNA can influence the expression of thousands of genes, thus controlling one-third of the human genome [3]. Recent studies have showed their association with human diseases and cancer [4, 5] and indicate that miRNA expression, whether intronic or intergenic, may be complex and varied among tissues, cell types, and disease states [6–8].

The promoter of a gene is a crucial control region for its transcription initiation [9, 10]. To make out the mechanism of the activation of miRNA genes, it is required to locate their core promoter regions. It has been noticed that promoter regions contain characteristic features that can be used to distinguish them from other parts of the genome. These features may be grouped into three types: signal, context, and structure features [11]. Signal features are biologically functional regions including core-promoter elements [11, 12], some short modular transcription factor binding sites (TFBSs), and CpG-islands [13], which play important roles in assembly of transcriptional machinery. Context features are extracted from the genomic content of promoters as a set of *n*-mers (*n*-base-long nucleotide sequences) whose statistics are estimated from training samples [14]. Structure features are derived from DNA three-dimensional structures, which play important roles in guiding DNA-binding proteins to target sites efficiently [15, 16].

However, only a small portion of the human miRNAs has confirmed transcription start sites (TSSs). Imperfect knowledge of the start sites of primary miRNA transcripts has limited our ability to study the promoter sequence features and further identify the transcription factor binding events. All existing promoter prediction methods for protein coding genes may not be suitable for miRNA genes, since they were not built based on the core promoters of miRNA genes. Hence, some studies have predicted the human pri-miRNAs boundary and regulatory region by EST [17, 18], sequence feature [19], and evolutionarily conservation [20]. Recently, several studies that utilized high-throughput genomic techniques identified the likely location of human miRNA TSSs [7, 8, 21–25]. The sequencing of 5′ transcript ends [26] and genome tilling microarrays (ChIP-chip) for RNA polymerase II [27] have been used to identify proximal promoters of miRNAs in* Arabidopsis*. Although numerous prediction models were developed for identifying miRNA promoters or TSSs, inadequate evidence was revealed to elucidate relationships between miRNA genes and transcription factors (TFs) due to lack of experimental validation.

With the next generation sequencing technology development, the genome-wide chromatin profiles have been detected. Using the combinations of specific histone modifications, chromatin states correlate with regulator binding, transcriptional initiation, and elongation; enhancer activity and repression can be inferred more precisely. Using biologically meaningful and spatially coherent combinations of chromatin marks, two studies have proposed a novel approach for discovering chromatin states, in a systematic de novo way across the whole genome based on a multivariate hidden Markov model (HMM) [28, 29] which explicitly modeled mark combinations. The chromatin marks included histone acetylation marks, histone methylation marks, and CTCF/Pol2/H2AZ. By analyzing the genome-wide occupancy data for these chromatin marks, the chromatin states were definitely classified. Even though states were learned de novo based solely on the patterns of chromatin marks and their spatial relationships, they correlated strongly with upstream and downstream promoters, 5′-proximal and distal transcribed regions, active intergenic regions, and repressed and repetitive regions. And these chromatin states were distinguished into six broad classes including promoter states, enhancer states, insulator states, transcribed states, repressed states, and inactive states according to the present/absent condition of the combination of chromatin marks.

Recently, genome-wide maps of nine histone modifications produced by ChIP-Seq were used to describe the chromatin patterns in* Arabidopsis* [30]. Previous study has found that miRNA and protein coding genes share similar mechanisms of regulation by chromatin modifications [31]. Based upon the assumption that the distributions of histone markers around the TSSs of miRNA genes are similar to the TSSs of protein coding genes, we have developed a computational strategy to identify the promoter regions of most miRNA genes. Comparing to HMM, the Support Vector Machine (SVM) has better performance in binary classification. In this study, SVM was used to distinguish the distributions of 9 histone markers in promoter regions and nonpromoter regions.

## 2. Method

### 2.1. Data Description

In the previous study, the ChIP-Seq experiments of nine histone modifications, H3K4me2, H3K4me3, H3K9Ac, H3K9me2, H3K18Ac, H3K27me1, H3K27me3, H3K36me2, and H3K36me3, were produced in the aerial tissue of 2-week-old* Arabidopsis* plants [30]. The whole genome profiles of nine ChIP-Seq experiments were downloaded from the National Center for Biotechnology Information Gene Expression Omnibus (accession number GSE28398). 35 bps color-space reads for each of the histone markers were aligned to TAIR 8* Arabidopsis thaliana* reference genome. Here, we converted the genome position of each read from TAIR 8 genome assembly to TAIR 10 genome assembly.

The gene annotation of TAIR 10 genome assembly was downloaded from* Arabidopsis* Information Resource (TAIR) (https://www.arabidopsis.org/), in which more than 27,000 protein coding genes were annotated.* Arabidopsis* miRNAs annotation was downloaded from miRNA registry [32] (miRBase, v20), and 298 miRNAs were annotated in miRBase.

### 2.2. ChIP-Seq Data Processing

In order to retrieve the histone modifications patterns around the transcription start sites of protein coding gene, we first divided the genomic regions neighboring TSS into 100 bp bins. We wanted to compare the same regions in the genome for the number of reads they have in nine different histone modification libraries, so we absolutely normalized the raw data to reads per million per bin (RPM). We calculated the number of reads that fall into an individual bin divided by the number of reads in the sample data set and then multiplied by 10^6^ to get the value per million. In this way we got an RPM track of 100 base bins covering the genome; thus samples with different numbers of reads become comparable. The RPM formula is as follows:

(1) $$\begin{array}{c} {\text{RPM}}_{i}=R_{i}*\frac{10^{6}}{N}. \end{array}$$

Here, *R*_*i*_ represents the number of reads falling into the *i*th bin and *N* represents the total number of mapped reads.

The histone binding pattern nearby the TSSs of all protein coding genes is retrieved as positive set, and the ones of 10,000 random positions are retrieved as negative set.

### 2.3. Support Vector Machine

In this study, the Support Vector Machine (SVM) was used to classify the TSSs and random regions based on the profiles of nine histone markers. The SVM is described as follows.

Given a training data *D*, a set of *n* points of the form

(2) $$\begin{array}{c} D={\left\{\left(x_{i},y_{i}\right)\;|\;x_{i}\in R,y_{i}\in \left\{-1,1\right\}\right\}}_{i=1}^{n}, \end{array}$$

where *x*_*i*_ is the reads count in each bin around the *i*th gene's TSS and the *y*_*i*_ is either 1 or −1, indicating the two classes to be classified as the real transcription start sites versus random genomic regions. 

The decision function of SVM is

(3) $$\begin{array}{c} \mathrm{sgn}\left(\overset{n}{\underset{i=1}{\sum }}y_{i}a_{i}K\left(x_{i},y_{i}\right)+\rho \right). \end{array}$$

Here, *K*(*x*_*i*_, *y*_*i*_) is the radial basis function (RBF) kernel, because of its good general performance and a few number of parameters (only two: *C* and *γ*).

We used the R package “e1017,” which offers an interface to package LibSVM (version 2.6). To obtain SVM classifier with optimal performance, the penalty parameter *C* and the RBF kernel parameter *γ* are tuned based on the training set using the grid search strategy in e1017.

## 3. Result 

### 3.1. The Histone Marker Distribution around TSS of Protein Coding Genes

**Figure 1:**
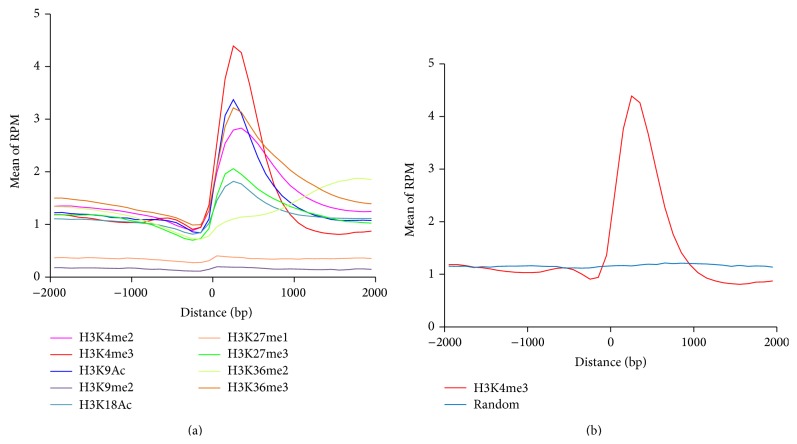
The distribution of histone markers around TSS of protein coding genes. (a) The ChIP-Seq-derived histone modifications patterns around the TSS of protein coding gene in* Arabidopsis*. The RPM distributions of nine histone markers including H3K4me2, H3K4me3, H3K9Ac, H3K9me2, H3K18Ac, H3K27me1, H3K27me3, H3K36me2, and H3K36me3 were marked by different colors. (b) The pattern of ChIP-Seq-derived H3K4me3 around the TSS of protein coding gene (red curve) and random genomic region (blue) in* Arabidopsis*.

The goal of this study was to use ChIP-Seq-derived histone marker data to identify transcription start sites of miRNAs in* Arabidopsis*. We first examined the 9-histone-marker pattern around the TSS of protein coding genes. We divided the genomic regions into multiple 100 bp bins and calculated the reads per million per bin (RPM) of each histone marker's fragments located in each of the bins within 2,000 bp upstream and downstream the TSS. Not surprisingly, most of these nine histone markers are enriched around the transcription start sites of protein coding genes and a peak of RPM can be found near the TSS (Figure 1(a)). While, among these nine histone markers, the H3K4me3 has the most significant peak, H3K9me2 and H3K27me1 have no peak in TSS. We also randomly selected 10000 genomic positions to examine the histone marker pattern. No such enrichment was found for H3K4me3 signal (Figure 1(b)) and other histone markers in random genomic regions. It suggests that the histone markers are strongly correlated with TSS. We can predict the promoter by examining the distribution of histone markers around TSS.

### 3.2. The Training and Prediction of SVM Using Nine Histone Markers

**Figure 2:**
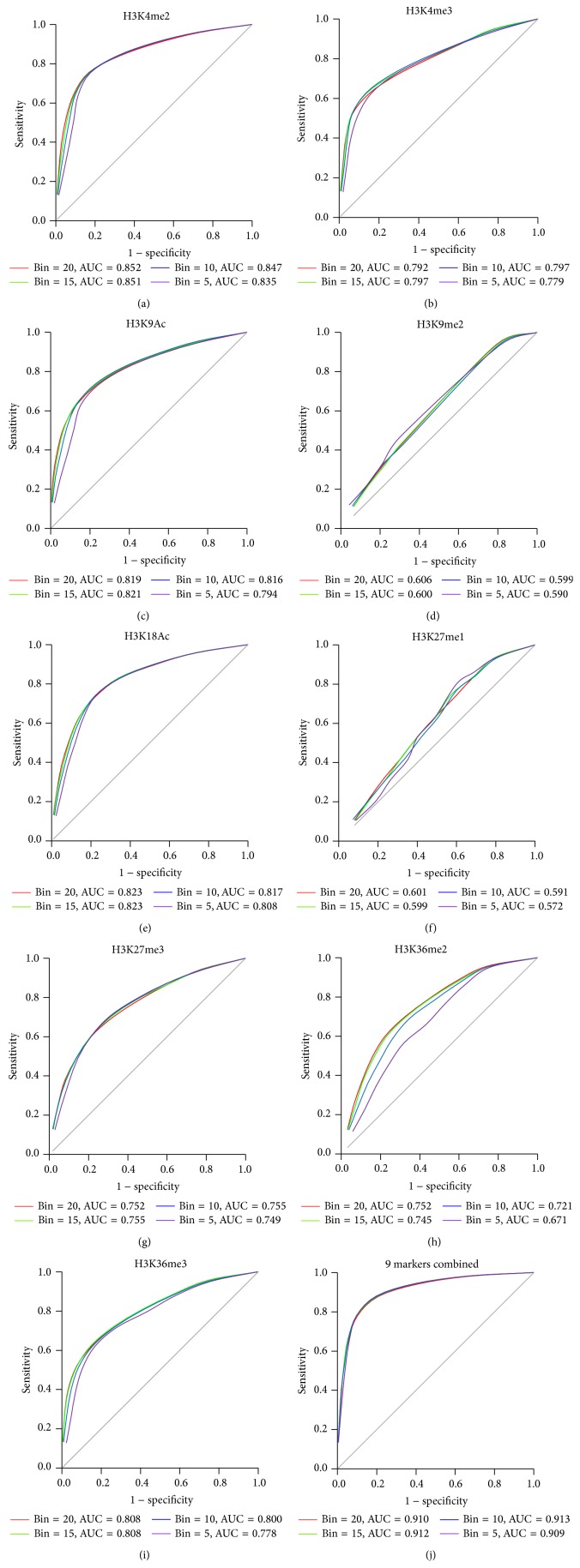
ROC curve for TSS prediction of protein coding genes with different histone markers. From (a) to (i), the ROC curve shows the sensitivity and specificity of the TSS prediction for protein coding genes with different histone marker. For each histone marker, the ROC curve was calculated within four different ranges around the TSS. For example, the red curve represents the ROC curve calculated within 20 bins up and 20 bins down of the TSS. The area under the curve (AUC) for each range around the TSS is shown in each graph. (j) The ROC curve for TSS prediction of protein coding genes with the combined nine histone markers.

In this study, we used Support Vector Machine (SVM) to predict the TSSs of miRNA based on the profiles of 9 histone markers. We selected the fragment distribution derived from ChIP-Seq data of 9 histone markers around TSS in 27,000 protein coding genes as positive set and those on 10,000 random positions as negative set. To estimate the accuracy of our method, we used random half of the positive set and half of the negative set to train SVM and then predicted the remaining positive and negative set. The prediction probability was presented using characteristic curve (ROC curve), in which the abscissa is specificity that represents the false positive rate and the ordinate is sensitivity that represents the true positive rate. If the area under ROC curve (AUC) is bigger, the accuracy of prediction is higher. At first, we used the distribution pattern of one single histone marker to train SVM and nine ROC curves of predicted result were shown in Figures 2(a)–2(i). For each histone marker, 4 different histone patterns were picked up around TSS, which were 20, 15, 10, and 5 bins up and down TSS. Notably, the H3K4me3 has the biggest AUC (~0.85) and the prediction accuracy in each pattern selection based on different bin number is very similar. On the contrary, the H3K9me2 and H3K27me1 have the smallest AUC (~0.6). This result is very consistent with the enrichment of histone marker pattern around the TSS. In most histone marker predictions, the AUC scores are increased from 5 bins to 20 bins, which means the statistical power is increased. To get more accurate prediction, we combined all the nine histone markers to train SVM and predict TSS (Figure 2(j)). All the AUC scores of 4 different histone patterns based on bin number are above 0.9. The highest AUC score is 0.913 based on prediction of 10 bins. In the next step, we will predict the miRNA transcription start sites by integrating 9 histone markers around 10 bins of upstream and downstream TSSs.

### 3.3. The Prediction of TSSs of miRNA Genes

**Figure 3:**
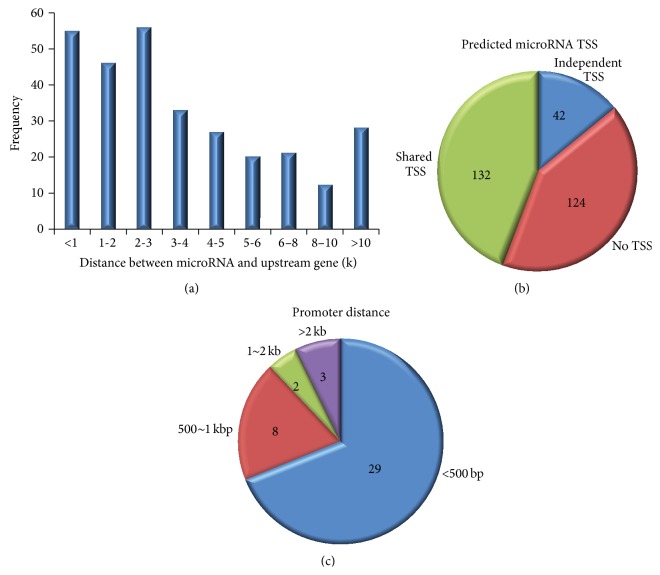
Features of predicted miRNA TSSs. (a) Histogram illustrating the distance between 298 miRNAs and their upstream genes. (b) The number of identified miRNA TSSs. The blue sector represents the 42 independent miRNA TSSs. The green sector represents the 132 predicted miRNA TSSs in the same position as the TSS of their upstream genes. The red sector represents 124 miRNAs that have no predicted TSS. (c) The distances between the predicted independent miRNA TSSs and their corresponding miRNAs.

The objective of this study was to identify the TSSs of miRNAs by searching for histone marker patterns similar to those seen in the upstream regions of protein coding genes. The* Arabidopsis* genome has a large density of gene distribution, which is about one gene every 4-5 kb. The distance between miRNA and the TSS of its nearest upstream gene was calculated (Figure 3(a)), which showed miRNAs whose corresponding distance is <1 k, 1-2 k, or 2-3 k had the highest frequencies. 217 of 298 distances between miRNAs and upstream TSSs are less than 5 k, and no miRNAs are far away from the nearest upstream TSS to 10 K. In this study, we focused our study on 298 miRNAs obtained from miRBase miRNA sequence database (version 20). For each miRNA, SVM was used to search the TSS of the primary miRNA up to 10 kbp upstream the mature miRNAs. We combined the profile of 9 histone markers in up and down 10 bins around TSS to train the SVM and then predict promoters of 298 miRNA genes. Using FDR ≤ 0.1, we identified 42 miRNAs which have independent TSSs (Table 1). Among 298 miRNAs, the predicted TSSs of 124 miRNAs were at the same position as the promoter of upstream nearest gene (Supplementary Table 1  in Supplementary Material available online at http://dx.doi.org/10.1155/2015/861402), and the remaining 132 miRNAs were not predicted (Figure 3(b)). We also calculated the distance between 42 independent TSSs and the corresponding miRNAs, which were shown in Figure 3(c). Obviously, most of the independent predicted promoters were much close to the corresponding miRNAs.

### 3.4. The Histone Pattern around Predicted miRNA TSSs

**Figure 4:**
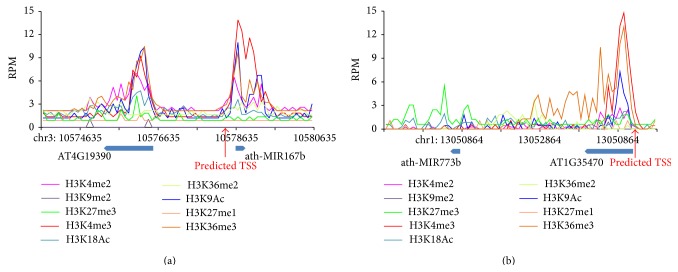
The histone pattern between predicted miRNA TSSs and upstream gene TSSs. (a) The example of independent predicted miRNA TSS. The first peak represents the position of the upstream gene TSS and the second peak represents the position of predicted miRNA TSS. (b) The example of predicted miRNA TSS has the same position as the TSS of upstream gene. Different histone markers are presented in different color.

miRNAs ath-MIR167b and ath-MIR773b are selected to show the histone pattern around the predicted TSSs (Figure 4). miRNA ath-MIR167b and upstream gene AT4G19390 are head-to-head gene pair (Figure 4(a)). Our method identified an independent TSS of ath-MIR167b, which is 2 k far away from gene AT4G19390. The histone marker profiles showed two different peaks of combined nine histone markers, which suggests these two opposite genes had differently regulatory regions. miRNA ath-MIR773b and upstream gene AT1G35470 are in the same strand (Figure 4(b)). The predicted TSS of ath-MIR773b overlaps with the AT1G35470 promoter region. Only one histone marker peak can be found in the promoter region of AT1G35470, and no histone marker is enriched between ath-MIR773b and AT1G35470. One biological mechanism is that the miRNAs are transcribed with the upstream genes at the same time. We found that 25 out of 124 miRNAs with the same TSSs as upstream genes were intronic miRNAs (Supplementary Table 1), which means these miRNAs had the same regulatory region as host genes.

### 3.5. The Comparison with Other miRNA Promoter Identification Methods

**Figure 5:**
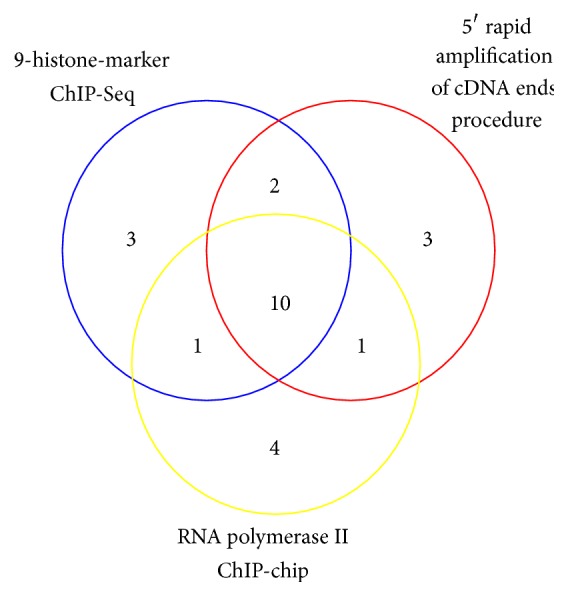
The overlapping of 16 microRNA TSSs identified by all three methods.

In previous studies, 5′ rapid amplification of cDNA ends procedure [26] and RNA polymerase II ChIP-chip experiment [27] have been used to determine promoters of miRNAs in* Arabidopsis*. Our study predicted 42 independent miRNA TSSs by 9 histone markers. 16 among 42 TSSs of miRNAs were also identified by other two methods. If a TSS position recognized by one method locates within 100 bp from TSS of the same miRNA identified by another method, this TSS was considered to be the same by these two methods. As we can see in Figure 5, 10 out of 16 miRNA TSSs were consistent for all three methods. One miRNA TSS was identified as being the same by our method and polymerase II ChIP-chip, but not by 5′ rapid amplification of cDNA ends procedure. Two miRNA TSSs were the same for our result and 5′ rapid amplification of cDNA ends procedure only.

## 4. Discussion

Annotation of the primary transcripts of miRNAs is extremely important to our understanding of the biological process of miRNAs and their regulatory targets. Although much progress has been made in promoter recognition, we are still far away from the goal of miRNA promoter identification. High-throughput DNA sequencing is rapidly changing the landscape of genomic research [33]. Recent studies using ChIP-Seq technology have revealed genome-wide transcription factor binding sites [34–36], RPol II binding sites and patterns associated with active transcription of coding genes [37, 38], and the distribution of histone modifications across the genome [38]. The modifications of the histones are found to be associated with transcription initiation and elongation [39], which made plenty of promoter prediction studies regarding histone modification as significant features. For example, H3K4me3 is enriched in the promoter regions, and H3K36me3 occurs at nucleosomes covering primary transcripts of actively expressed genes [40].

In this study, 9 histone markers including H3K4me2, H3K4me3, H3K9Ac, H3K9me2, H3K18Ac, H3K27me1, H3K27me3, H3K36me2, and H3K36me3 were used to predict miRNA promoters. Based upon the assumption that the distributions of histone markers around the TSSs of miRNAs are similar to the ones of protein coding genes, we developed a computational strategy to identify the promoter regions of all miRNA genes in* Arabidopsis*. Integrating 9 histone markers profiles, the model based on SVM classifier identified 42 independent miRNA TSSs from total 298 miRNA genes, and 132 predicted miRNA TSSs were identified in the same position as the TSS of their upstream genes. We also found that 25 out of 124 miRNAs were intronic miRNAs, which suggest that most of the intronic miRNAs share promoter regions with their host genes. For the remaining genes, we lack the evidence to explain whether they share the promoter with the upstream genes or have independent promoter. So the identification of miRNA promoters in other tissues of* Arabidopsis*, in addition to the aerial tissue, will improve the annotation of primary transcripts of miRNAs.

## Supplementary Material

The additional file provides 132 predicted TSSs of miRNAs which are overlapping with TSSs of upstream nearest genes.

## Figures and Tables

**Table 1:** 42 independent predicted miRNA transcription start sites.

Index	miRNA ID	miRNA name	Genome coordinates	TSS
1	MI0000989	ath-MIR171b	chr1:3961348–3961464(−)	3961764–3961864
2	MI0005386	ath-MIR830	chr1:4820355–4820549(−)	4820549–4820649
3	MI0000218	ath-MIR159b	chr1:6220646–6220841(+)	6220446–6220546
4	MI0001005	ath-MIR394a	chr1:7058194–7058310(+)	7055994–7056094
5	MI0019201	ath-MIR5630a	chr1:12011152–12011223(−)	12011523–12011623
6	MI0019211	ath-MIR5630b	chr1:12023526–12023597(−)	12023997–12024097
7	MI0000193	ath-MIR161	chr1:17825685–17825857(+)	17825485–17825585
8	MI0019208	ath-MIR5636	chr1:18549959–18550036(+)	18549659–18549759
9	MI0001078	ath-MIR406	chr1:19430078–19430277(−)	19431177–19431277
10	MI0019235	ath-MIR5652	chr1:23412989–23413436(−)	23413636–23413736
11	MI0000196	ath-MIR163	chr1:24884066–24884396(+)	24883966–24884066
12	MI0001425	ath-MIR414	chr1:25137456–25137563(−)	25137763–25137863
13	MI0000189	ath-MIR159a	chr1:27713233–27713416(−)	27713616–27713716
14	MI0015817	ath-MIR4228	chr1:28889375–28889532(+)	28889175–28889275
15	MI0005105	ath-MIR775	chr1:29422452–29422574(+)	29422052–29422152
16	MI0001013	ath-MIR396a	chr2:4142323–4142473(−)	4142673–4142773
17	MI0005109	ath-MIR779	chr2:9560761–9560923(+)	9560161–9560261
18	MI0020189	ath-MIR5995b	chr2:10026910–10027050(+)	10026310–10026410
19	MI0020188	ath-MIR5595a	chr2:10026910–10027050(−)	10027050–10027150
20	MI0000178	ath-MIR156a	chr2:10676451–10676573(−)	10676673–10676773
21	MI0000215	ath-MIR172a	chr2:11942914–11943015(−)	11943215–11943315
22	MI0017889	ath-MIR5021	chr2:11974711–11974881(−)	11975181–11975281
23	MI0000201	ath-MIR166a	chr2:19176108–19176277(+)	19176008–19176108
24	MI0001072	ath-MIR403	chr2:19415052–19415186(+)	19414952–19415052
25	MI0000208	ath-MIR167a	chr3:8108072–8108209(+)	8107972–8108072
26	MI0005383	ath-MIR827	chr3:22122760–22122936(−)	22123036–22123136
27	MI0000202	ath-MIR166b	chr3:22922206–22922325(+)	22921906–22922006
28	MI0002407	ath-MIR447a	chr4:1528134–1528370(−)	1529270–1529370
29	MI0017896	ath-MIR5026	chr4:7844496–7844688(+)	7842896–7842996
30	MI0005405	ath-MIR850	chr4:7845707–7845927(+)	7842907–7843007
31	MI0005440	ath-MIR863	chr4:7846597–7846899(+)	7842897–7842997
32	MI0015815	ath-MIR4221	chr4:8460516–8460662(+)	8459516–8459616
33	MI0000210	ath-MIR168a	chr4:10578635–10578772(+)	10578335–10578435
34	MI0000180	ath-MIR156c	chr4:15415418–15415521(−)	15415821–15415921
35	MI0019242	ath-MIR5658	chr4:18485438–18485531(−)	18486431–18486531
36	MI0000198	ath-MIR164b	chr5:287584–287736(+)	287484–287584
37	MI0000216	ath-MIR172b	chr5:1188207–1188301(−)	1188501–1188601
38	MI0000195	ath-MIR162b	chr5:7740598–7740708(−)	7740908–7741008
39	MI0019216	ath-MIR5643a	chr5:11667797–11667879(+)	11667197–11667297
40	MI0001014	ath-MIR396b	chr5:13611798–13611932(+)	13611698–13611798
41	MI0000211	ath-MIR168b	chr5:18358788–18358911(−)	18359011–18359111
42	MI0001075	ath-MIR405b	chr5:20632514–20632637(+)	20630514–20630614
